# Tenascin-C is a driver of inflammation in the DSS model of colitis

**DOI:** 10.1016/j.mbplus.2022.100112

**Published:** 2022-05-23

**Authors:** James Ozanne, Brandon Shek, Louise A. Stephen, Amanda Novak, Elspeth Milne, Gerry Mclachlan, Kim S. Midwood, Colin Farquharson

**Affiliations:** a The Roslin Institute, Royal (Dick) School of Veterinary Studies, University of Edinburgh, United Kingdom; b Royal (Dick) School of Veterinary Studies, University of Edinburgh, United Kingdom; c Kennedy Institute of Rheumatology, Nuffield Department of Orthopaedics, Rheumatology and Musculoskeletal Sciences, Oxford University, United Kingdom

**Keywords:** Tenascin-C, Inflammatory Bowel Disease, Colitis, Inflammation

## Abstract

Inflammatory Bowel Disease (IBD) is a grouping of chronic inflammatory disorders of the gut. Tenascin-C is a pro-inflammatory, extracellular matrix protein found upregulated in IBD patients and whilst a pathological driver of chronic inflammation, its precise role in the etiology of IBD is unknown. To study tenascin-C’s role in colitis pathology we investigated its expression in a murine model of IBD. Wild-type (WT) or tenascin-C knockout (KO) male mice were left untreated or treated with dextran sodium sulphate (DSS) in their drinking water. Tenascin-C was upregulated at the mRNA level in the colitic distal colon of day eight DSS treated mice, coinciding with significant increases in gross and histological pathology. Immunohistochemistry localized this increase in tenascin-C to areas of inflammation and ulceration in the mucosa. Tenascin-C KO mice exhibited reduced gross pathology in comparison. These differences also extended to the histopathological level where reduced colonic inflammation and tissue damage were found in KO compared to WT mice. Furthermore, the severity of the distal colon lesions were less in the KO mice after 17 days of recovery from DSS treatment. This study demonstrates a role for tenascin-C as a driver of inflammatory pathology in a murine model of IBD and thus suggests neutralizing its pro-inflammatory activity could be explored as a therapeutic strategy for treating IBD.

## Highlights

• Increased tenascin-C staining appeared to predominantly occur in damaged ulcerated areas.
• Tenascin-C knock-out mice were partly protected from DSS induced colitis.
• Mice deficient in tenascin-C had areas of + ve EpCAM staining indicating that crypt and epithelial integrity was maintained.

## Introduction

Inflammatory Bowel Disease (IBD) encompasses a group of conditions in which an aberrant chronic inflammatory response of unknown etiology targets the intestinal tract, resulting in its progressive dysfunction and destruction [1]. IBD presents clinically as two main subtypes: ulcerative colitis and Crohn’s disease. Ulcerative colitis is characterized by continuous mucosal inflammatory lesions affecting only the colon whereas Crohn’s disease presents as patchy transmural inflammation potentially affecting any region along the alimentary tract, although most commonly found in the distal small intestine [2]. Both of these conditions are chronic with no medically curative therapies available except for major surgical intervention, with its own potentially serious complications, in extreme cases of ulcerative colitis [3]. This is particularly problematic when considering disease burden has been increasing globally in recent decades with prevalence now surpassing 0.3% in most of Europe and North America [4]. Further work is needed to understand the pathogenesis and drivers of the disease and to aid in the development of effective tools for its clinical management and treatment.

Tenascin-C is an extracellular matrix (ECM) protein that was first described in the 1980s, and together with tenascin-R, tenascin-W, and tenascin-X make up the tenascin family of proteins [5]. Tenascin-C is expressed dynamically at numerous sites during embryonic development, including during epithelial morphogenesis [6], neurogenesis [7], and developing connective tissues [8]. In the adult this expression becomes more sparse and restricted, narrowing to a few tissues under tensile stress, such as muscle [9], underlying some epithelia, such as blood vessels [10], and within a number of stem cell niches [11].

Whilst this basal expression pattern attributed a variety of functions to tenascin-C during development its expression in response to inflammation has resulted in tenascin-C being implicated as a pathological driver of chronic inflammation. This has been demonstrated in a variety of different tissues, and in response to a wide range of insults [12]. In these scenarios tenascin-C acts as a damage associated molecular pattern (DAMP), a class of endogenous molecules which promote and drive inflammation through their interactions with immune and stromal cells [13]. In the case of tenascin-C this interaction has been shown to be mediated by the pattern recognition receptor (PRR) Toll-Like Receptor 4 (TLR4) [14] as well as the integrins α9β1 [15] and αVβ3 [16], whose engagement by tenascin-C promotes cell activation and often the production of a variety of pro-inflammatory mediators. While this pro-inflammatory action may be beneficial in the normal physiological response to injury it has become increasingly appreciated that DAMPs also play a role in the pathogenesis of inflammatory disease [17]. In this context, tenascin-C itself is implicated in a diverse range of chronic inflammatory conditions such as rheumatoid arthritis [18], systemic lupus erythematosus [19], as well as IBD [20], [21]. Tenascin-C’s deposition at sites of inflammation and its ability to drive pro-inflammatory signaling pathways suggest it could function to create a pro-inflammatory niche at sites of disease [22]. This microenvironment created by tenascin-C helps propagate the local inflammatory response by forming a persistent pro-inflammatory positive feedback loop. [23], [24]. In this manner, tenascin-C plays a key role in the deregulated inflammatory response by blocking resolution and aiding its propagation to chronicity. This ultimately leads to tissue succumbing to dysfunction and destruction as witnessed in chronic inflammatory diseases such as IBD.

Systemic levels of circulating tenascin-C are increased in IBD patients compared to healthy controls [25]. Furthermore, these levels correlate with disease activity and disclose responsiveness to therapy with proctocolectomy surgery and successful biologic therapy, resulting in a significant decrease in tenascin-C levels [26]. This systemic increase in IBD is also reflected at the local level with a number of histological studies reporting high levels of tenascin-C staining in the inflamed gut tissue of patients with ulcerative colitis [27], Crohn’s disease [28], and microscopic colitis [29]. However, whilst these studies demonstrate an association between IBD and tenascin-C, the exact role tenascin-C might be playing in IBD etiology has yet to be fully investigated. Therefore, to advance previous RNA expression studies we have in this study used the dextran sodium sulphate (DSS) colitis model to examine the spatiotemporal expression of tenascin-C during the development of murine colitis. Furthermore, to determine any contribution of tenascin-C in driving the inflammation seen in this model we utilized a tenascin-C knockout (*Tnc^−/−^)* mouse, which in combination with the DSS model allowed the dissection of tenascin-Cs contribution to pathological changes in colitis.

## Materials and methods

### Animals

**Fig. 1:**
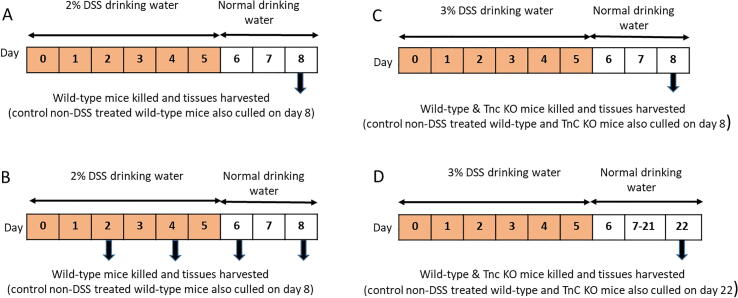
Experimental design of the various DSS studies. (A) DSS and control wild-type mice culled at day 8 (B) DSS and control wild-type mice culled at two-day intervals up to day 8 (C) DSS and control wild-type and Tnc KO mice culled at day 8 (D) DSS and control wild-type and Tnc KO mice culled at day 22.

Male 7–9 week old wild-type (WT) and *Tnc^−^*^/^*^−^* 129sv mice were used for all experiments and were maintained under specific pathogen free conditions in individually ventilated caging at the Roslin Institute (University of Edinburgh, UK) with a 12-hour light/dark cycle. *Tnc^−^*^/^*^−^* mice were generated as previously described [30]. All animal experiments were approved by the Roslin Institute’s named veterinary surgeon and named animal care and welfare officer (NACWO), with animals maintained in accordance with the Home Office code of practice (for the housing and care of animals bred, supplied or used for scientific purposes). Colitis grade 36,000–50,000 MW DSS (MP Biomedicals, Santa Ana, USA) was administered in the drinking water *ad libitum* at a concentration of 2 or 3% (w/v) for five days. DSS was then withdrawn and the mice returned to regular drinking water for the remaining period before cull. In the acute model the mice were left for 3 days following DSS withdrawal and in the recovery model the mice were left for 17 days [31] (Fig. 1). Age and sex matched control mice.

received normal drinking water for the duration of the experiment. To control for potential genotypic differences in water consumption between *Tnc^−^*^/^*^−^* and WT mice, water intake (of all mice in 1 cage) was monitored during the DSS dosing period. The body weight and health status of the DSS-treated mice were scored daily and this involved monitoring for changes in appearance such as coat staring, hunching, and change in gait; and behavioural changes such as reluctance to move, and restlessness. Additionally, colitis specific changes in stool consistency and presence of fecal blood were also scored. A daily disease activity index (DAI) score for each mouse was produced by summing these scoring parameters (Supplementary Table 1). Mice that met DAI scoring severity thresholds, and/or lost more than 20% body weight before the study end point were euthanized on humanitarian grounds in compliance with home office regulations and were not included in the analysis. At the endpoint of all experiments the mice were culled by exsanguination by cardiac puncture while under terminal isoflurane induced anaesthesia to provide a terminal blood sample from which serum was obtained and stored at −70 °C.

### Tissue collection

Colons were transected at the colorectal and cecocolonic margins and resected with as much mesenteric tissue removed as possible. After flushing with phosphate buffered saline (PBS) the colon length was measured and transected into 3 equal segments corresponding to the proximal, middle, and distal portions of colon. These segments were further subdivided and processed for RNA extraction, histology and cryotomy.

### Colon histopathology grading

Colon segments were flushed with 10% neutral buffered formalin (NBF; CellPath, Powys, UK), then placed in histology cassettes (Simport, Beloeil, Canada) and incubated in 10% NBF for a minimum of 48hrs with agitation at 4 °C. Cassettes were transferred and stored in 70% ethanol and processed through to wax following standard procedures. Colon segments were embedded transversely, sectioned at 5 µm, mounted on Superfrost slides (Thermo Fisher Scientific) and finally stained with H&E. Histopathological grading was performed blind using a published colitis grading system [32], which had been used previously in our laboratory [33]. Pathology was scored for at least one section of proximal, middle, and distal colon per mouse. The scoring system assessed inflammation severity, inflammatory extent through the tissue, tissue regeneration, and crypt damage. Additionally, a grade was also given for each of these parameters based on the percentage of the tissue, which was affected by it. Final scores for the individual scoring parameters were calculated as the parameters score multiplied by its percentage involvement grade (Table 1). Sum histopathology scores were calculated as the sum of each of these individual scoring parameters. Data for individual parameters as well as the sum score are presented as median score ± interquartile range.

**Table 1:** Scheme used for colitis histopathology grading.

Feature Graded	Grade	Description
Inflammation	0	None
	1	Slight
	2	Moderate
	3	Severe

Extent	0	None
	1	Mucosa
	2	Mucosa and submucosa
	3	Transmural

Regeneration	4	No tissue repair
	3	Surface epithelium not intact
	2	Regeneration with crypt depletion
	1	Almost complete regeneration
	0	Complete regeneration or normal tissue

Crypt Damage	0	None
	1	Basal 1/3 damaged
	2	Basal 2/3 damaged
	3	Only surface epithelium intact
	4	Entire crypt and epithelium lost

Percentage Involvement	1	1–25%
	2	26–50%
	3	51–75%
	4	76–100%

### Immunohistochemistry

Colon segments were covered with Optimal Cutting Temperature (OCT) embedding compound (Cellpath) and snap frozen in an ethanol-dry ice slurry cooled hexane bath [34]. Cryosections were cut in a transverse orientation at 7 µm on an OTF5000 cryostat (Bright Instruments, Bedfordshire, UK), mounted and air dried before storage at −70 °C. Sections were washed with PBS-tween 20 (PBST; 0.05%) before blocking and the addition of primary antibodies overnight at 4 °C in a humidity chamber. The primary targets were, Tenascin-C, CD3, CD11c, CD326 (EpCAM), Lyve-1 and Collagen type IV. Slides were washed with PBST before application of fluorescent or biotin conjugated secondary antibodies for 1 h at room temperature. Slides which were incubated with biotinylated secondary antibodies were washed with PBST and incubated with streptavidin conjugated to AF594 for 1 h. All antibodies used are listed in Supplementary Tables 2 and 3. All slides were then counterstained by incubation with DAPI (Thermo Fisher Scientific) nuclear stain at 0.1 µg/ml in PBS for 5 min. The slides were finally cover-slipped using ProLong Gold Antifade Mountant (Thermo Fisher Scientific) before imaging. Fluorescently stained sections were visualised using a Leica DMLB upright fluorescent microscope fitted with a Coolsnap MYO low light monochrome CCD camera (Teledyne Photometrics, Tuscon, USA). For control sections, the primary antibody was replaced with non-specific IgG from the animal species in which the primary antibody was raised.

### RNA extraction and RT-qPCR

Colon tissue was flash frozen in LN_2_ and stored at −70 °C until use. Tissue (∼20 mg) was homogenised in QIAzol lysis buffer (Qiagen, Hilden, Germany) and 200 µl of chloroform was added before 15 s vigorous mixing. Phases were then separated by centrifugation at 12,000 rpm for 15 min at 4 °C after which the upper aqueous phase was taken off and transferred to a separate tube. RNA was purified using an RNeasy spin column kit (Qiagen) following the manufacturer’s instructions. RNA concentration and purity was assessed using a NanoDrop 1000 spectrophotometer (Thermo Fisher Scientific, Waltham,MA, USA) checking that the A260/A280 and the A260/230 ratios were both approximately 2.0 indicating low protein and organic compound contamination, respectively. RNA was reverse transcribed using SuperScript II (Thermo Fisher Scientific) as per the manufacturer’s instructions. cDNA samples were stored at −20 °C until used, Gene expression using cDNA diluted to 5 ng/µl was assayed using Primer Design PrecisionPlus Master Mix with premixed SYBR Green (PCR BioSystems, London, UK), using a MX3000P qPCR system (Stratagene, San Diego, USA). Gene expression data were normalized to 18S and analysed using the ΔΔCt method [35]. Oligonucleotide primers were obtained from Sigma-Aldrich or Primer Design (Supplementary Table 2).

### Statistical analysis

Statistical analysis was carried out using Prism 8 statistical software (Graphpad Software, San Diego, USA). Normally distributed continuous data including body weight loss and colon length were analysed using Student’s *t*-test, a one-way ANOVA, or a two-way ANOVA with Sidak’s multiple comparisons test. Non-normally distributed continuous data or ordinal data, such as DAI and histopathology scoring, were analysed using the appropriate non-parametric Mann-Whitney U or Kruskal-Wallis with Dunn’s multiple comparisons tests. P < 0.05 was considered to be significant for all tests. All continuous data are presented as the mean ± the standard deviation (StdDev) and ordinal data as the median ± the interquartile range.

## Results

**Fig. 2:**
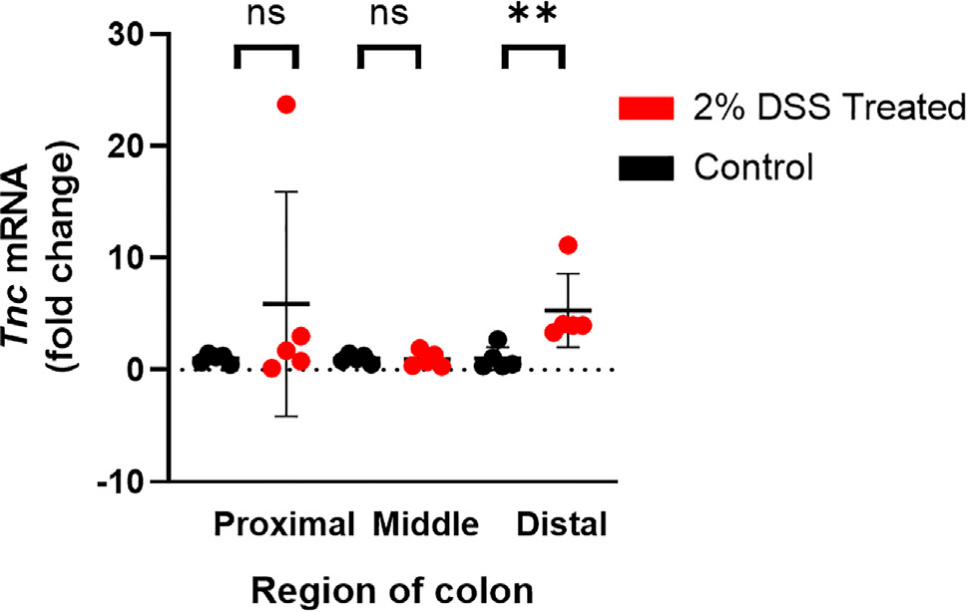
Tenascin-C expression in colon segments of control and DSS treated mice. RT-qPCR analysis of tenascin-C (*Tnc*) gene expression in the proximal, middle and distal colon of control and DSS treated treated mice. Data presented as mean fold change compared to matching control region ± SD, n = 4–5 mice/group. ** p < 0.01, ns = not significant.

In a preliminary study (Fig. 1a), tenascin-C mRNA expression was increased in the distal colon of DSS treated mice compared to controls at day 8. No significant difference in tenascin-C mRNA expression between the groups was observed in either the proximal or middle colon (Fig. 2).

**Fig. 3:**
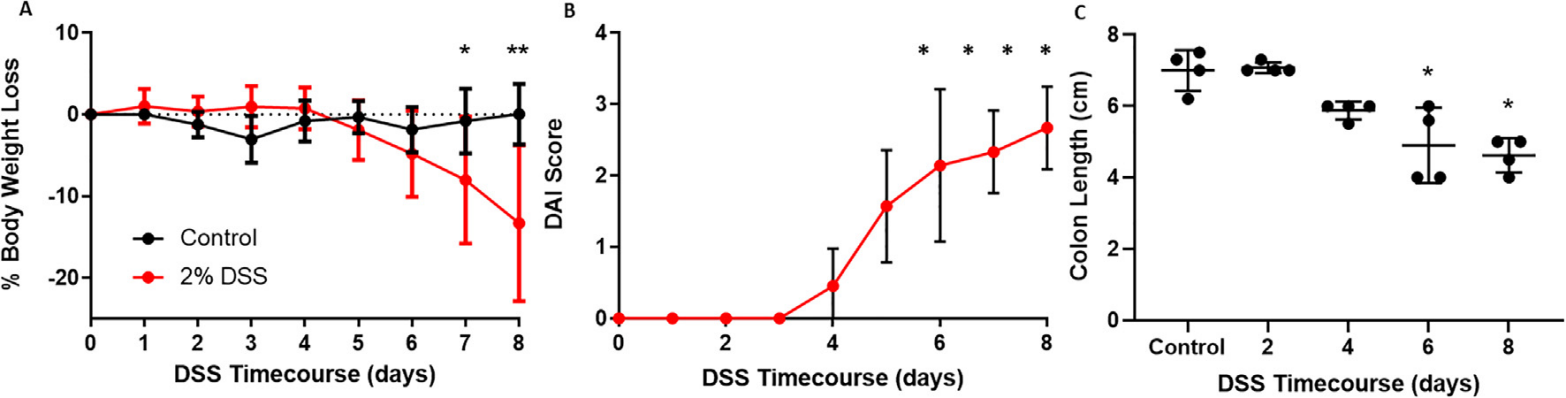
DSS colitis gross pathology. (A) Body weight of control and DSS treated mice over the 8 day experimental period. Data presented as mean body weight loss compared to day 0 ± SD, n ≥ 3 mice/group at each time point. (B) Disease activity index (DAI) of DSS treated mice. No scoring was observed for control mice. Data presented as median ± interquartile range, n ≥ 4 per time point, significance compared to day 0. (C) Colon length of control and DSS treated mice over the 8 day experimental period. Data presented as mean ± SD, n = 4 mice/group at each time point. * p < 0.05; ** p < 0.01.

Having demonstrated tenascin-C’s increased expression at the acute stage of inflammation we next interrogated the temporal nature of this increase at the protein level. To do this we completed a DSS time course experiment in which mice were culled at two-day intervals up to the study end-point at day 8 (Fig. 1b). During the DSS treatment period (0–5 days), no weight loss was observed but after then weight loss was rapid and reached significance at day 7 (p < 0.05) and day 8 (p < 0.001) (Fig. 3a). The DAI scoring scheme showed the development of symptoms, such as the presence of pasty stools and fecal blood, preceding the weight loss starting from as early as day four and becoming significantly established at day five (Fig. 3b). Finally, colon length at cull (day 8), demonstrated a trend towards decreasing length which became significant from day six (p < 0.05) (Fig. 3c).

**Fig. 4:**
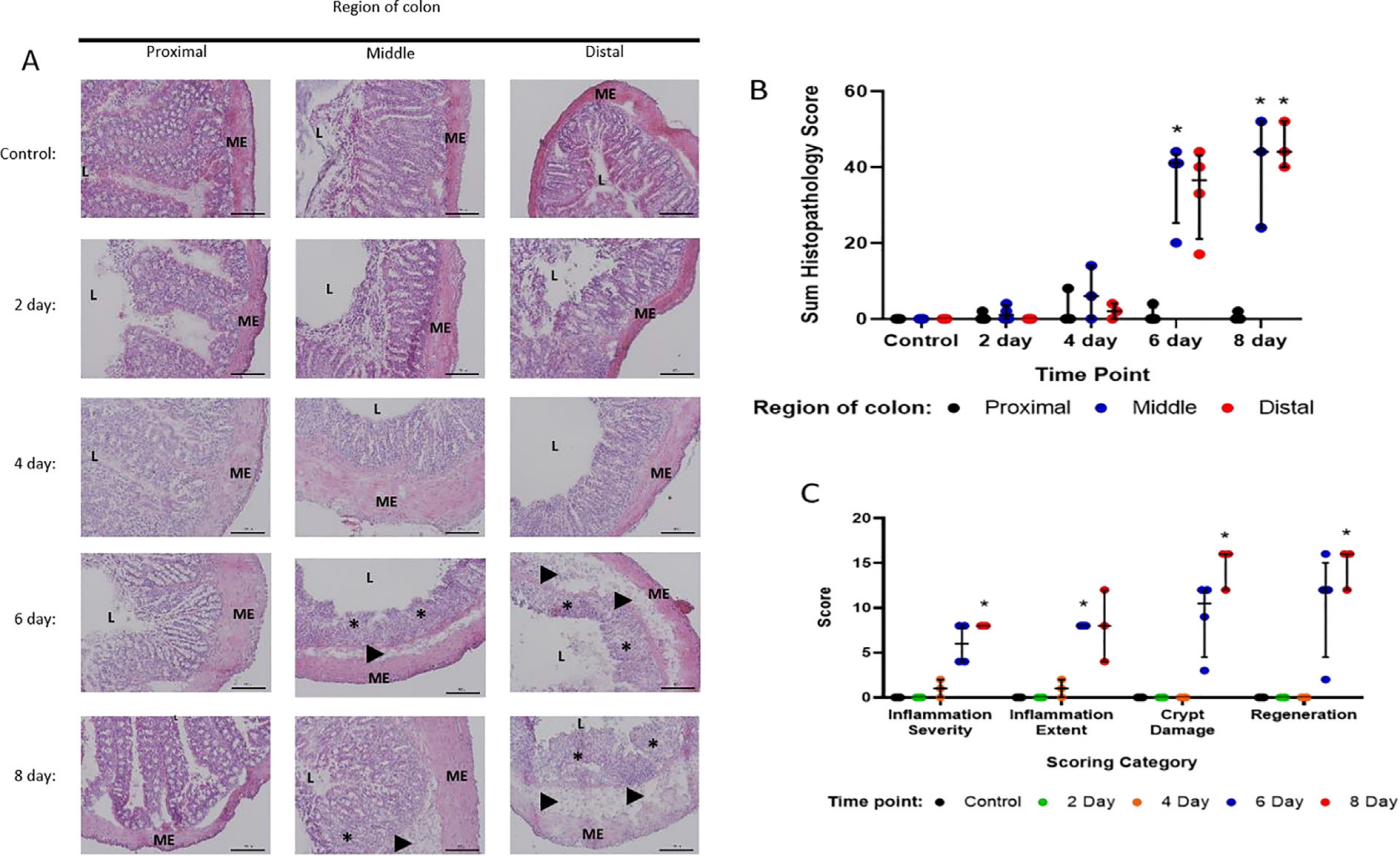
Histopathology scoring of H&E stained colon sections from DSS colitis time course mice. (A) H&E stained proximal, middle, and distal colon sections from control and DSS treated mice throughout the 8 day time course. Mixed inflammatory cell infiltration of the mucosa (arrows), submucosal edema (arrowheads) and ulceration (asterisks) are indicated. Scale bars = 100 µm. ME = muscularis externa, L = lumen. (B) Histopathology scores for proximal, middle, and distal colon sections from control and DSS treated mice throughout the 8 day time course. (C) Histopathology scores for individual scoring parameters in distal colon segments from control and DSS treated mice throughout the 8 day time course. Data points represent scores for individual mice with bars marking the median ± interquartile range, n = 3–4 per group, * p < 0.05.

Histological analysis of H&E stained sections indicated that at day 2 the histology along the length of the DSS colon appeared normal and was indistinguishable from control mice. From day four of DSS treatment the middle and distal colon showed some minor infiltration of mononuclear inflammatory cells, but tissue architecture appeared normal. This dramatically changed at days 6 and 8 where submucosal edema and inflammatory cell infiltration, mainly neutrophils as well as occasionally mononuclear cells were noted in the middle and distal colon. Additionally, disrupted mucosal architecture with extensive ulceration, loss of crypts, and hyperplasia, was now also readily apparent (Fig. 4a). These changes were quantified, confirming that tissue damage was only apparent from day 6 onwards (Fig. 4b). Breakdown by scoring parameter for the distal colon further supported earlier observations that the early pathological changes seen at day four was due solely to inflammatory changes. Tissue damage conversely only began to appear at day six (Fig. 4c).

**Fig. 5:**
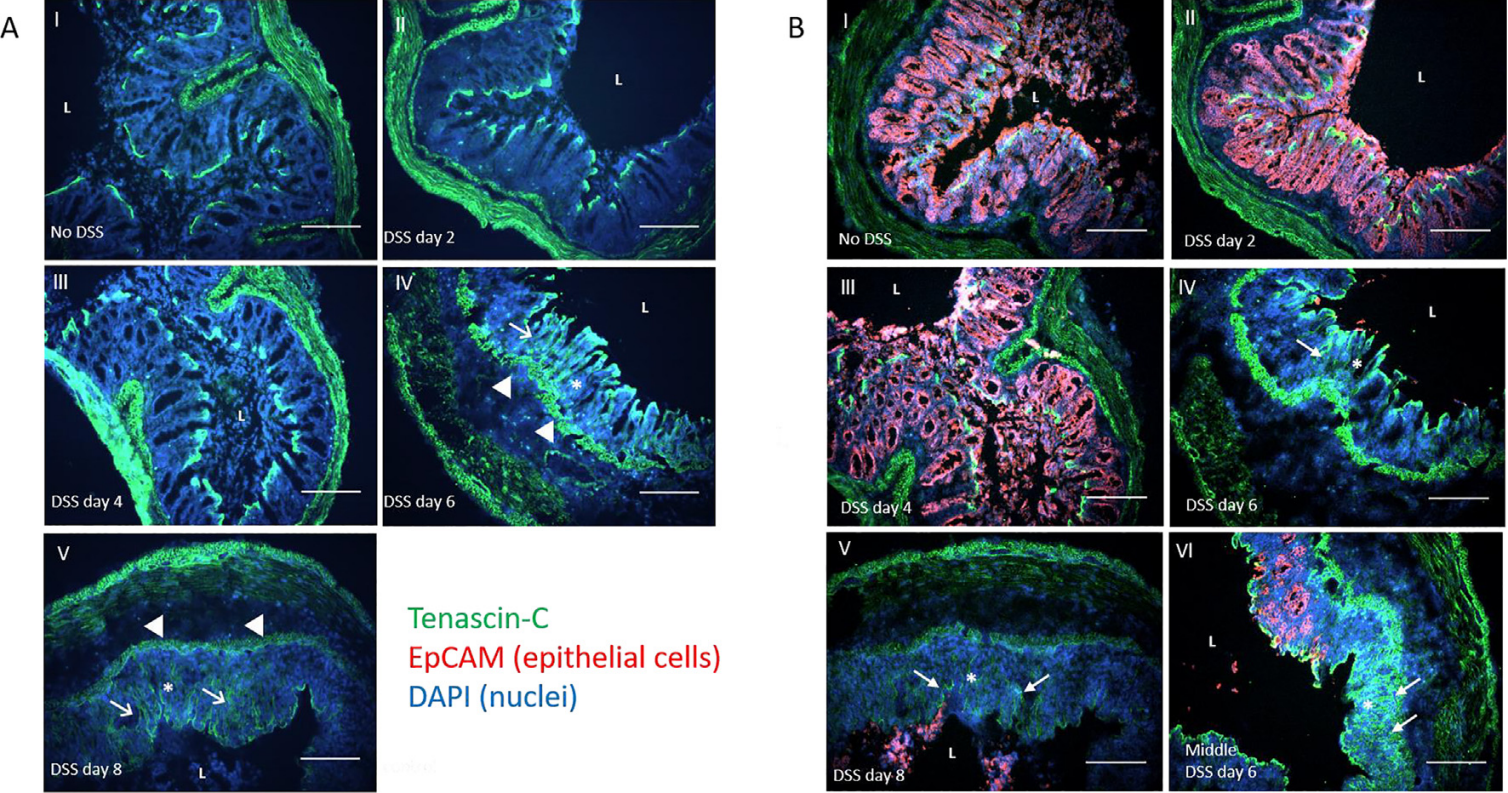
DSS induced mucosal tenascin-C expression localizes to areas of ulceration. (A) Immunohistochemical staining for tenascin-C (green) of distal colon sections from control and DSS treated mice throughout the 8 day time course. Mixed inflammatory cell infiltration and submucosal edema (arrowheads), ulceration (asterisks), mucosal hyperplasia (arrows) are indicated. (B) Immunohistochemical dual staining for tenascin-C (green) and EpCAM (red) of middle and distal colon sections from control and DSS treated mice throughout the 8 day time course. EpCAM strongly stains the colonic epithelium under basal conditions. Ulceration, shown by loss of EpCAM mucosal staining (asterisks), is present from day six in the DSS treated mice, with tenascin-C appearing to localise predominantly to these areas of damage (arrows). All slides were counterstained with the nuclear stain DAPI (blue). Scale bars = 250 µm. L = lumen. (For interpretation of the references to colour in this figure legend, the reader is referred to the web version of this article.)

Tenascin-C protein expression and distribution was determined within the distal colon by immunohistochemistry. This approach revealed that in normal tissue tenascin-C was restricted to muscularis externa, as well as a sub-epithelial band in the lamina propria directly underlying the surface epithelium. (Fig. 5ai). At days 6 and 8 in DSS treated mice, tenascin-C expression had an altered and more widespread distribution than control tissue where it was more prominent in areas with heightened damage (Fig. 4a iv, v). The localization of tenascin-C appeared normal at days 2 and 4 (Fig. 5a ii, iii). The apparent increased tenascin-C expression in areas of mucosal damage was further investigated in sections double stained for tenascin-C and the epithelial cell marker, EpCAM. The mucosal epithelium stained strongly for EpCAM along the length of the colon in control tissue (Fig. 5b i). A similar staining pattern was observed in the proximal colon throughout the induction of colitis corroborating the observed lack of tissue damage in this region (data not shown). For the middle and distal colon however ulceration, evidenced by loss of EpCAM mucosal staining, was observed which exposed the underlying connective tissue and subepithelial tenascin-C to the lumen. In some cases, the epithelial debris from the sloughed epithelium was also present in the colonic lumen overlying these areas (Fig. 5b iv, v). In terms of colitis induced tenascin-C staining this appeared to predominantly occur in these damaged ulcerated areas. In contrast, areas maintaining at least some EpCAM expression showed reduced or absent mucosal tenascin-C staining suggesting tenascin-C was being deposited directly at the sites of mucosal injury (Fig. 5b vi).

**Fig. 6:**
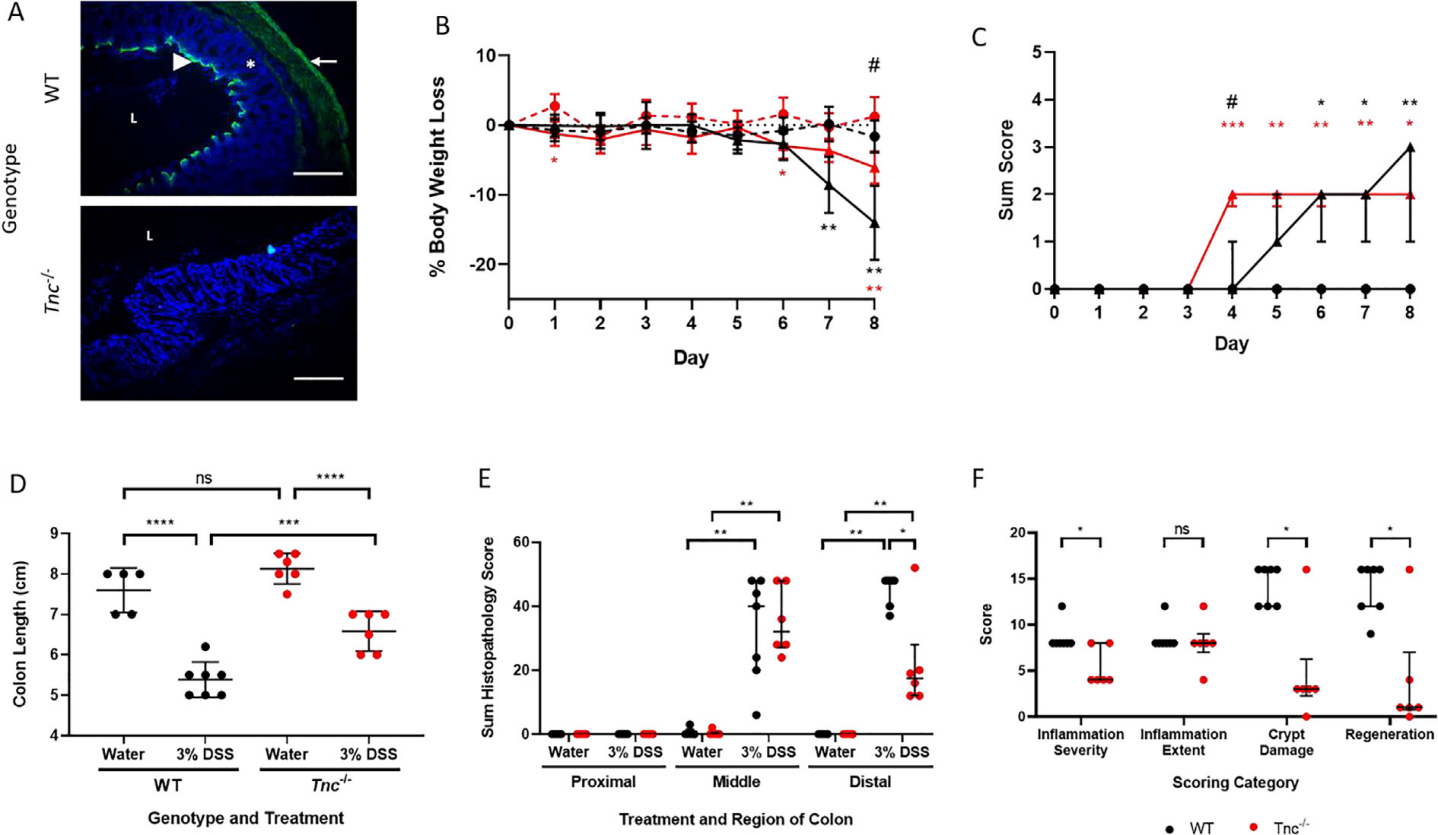
*Tnc^−^*^/^*^−^* mice show reduced DSS colitis gross pathology compared to wild-type (WT) controls. (A) Immunohistochemical staining for tenascin-C (green) of distal colon sections from WT and *Tnc^−^*^/^*^−^* mice. Staining is observed in WT mice in the muscularis externa (arrow), in a subepithelial band (arrowhead), and in the muscularis mucosa (asterisk) along the length of the colon. *Tnc^−^*^/^*^−^* mouse tissue shows no staining confirming genetic ablation of the protein. Sections were counterstained with the nuclear stain DAPI (blue). Scale bars = 250 µm. L = lumen. (B) Percentage body weight loss compared to day 0 of WT (black line) or *Tnc^−^*^/^*^−^* (red line) mice offered normal drinking water (hatched line) or drinking water supplemented with DSS (intact line). Data presented as mean ± SD, n = 5–7 mice/group. Significance compared to genotype matched controls shown with asterisks, * p < 0.05; ** p < 0.01. # = p < 0.05 for DSS *Tnc^−^*^/^*^−^* compared to DSS WT. (C) Disease activity index (DAI) scoring data. Both genotypes showed a significant increased score by day 8 upon DSS dosing. No scoring was observed for control mice. Data presented as median ± interquartile range, n = 5–7 mice/group. Significance compared to genotype matched controls shown with asterisks, * p < 0.05; ** p < 0.01; *** p < 0.01. # = p < 0.05 for DSS *Tnc^−^*^/^*^−^* compared to DSS WT. (D) Colon length at cull on day 8. Data presented as individual data points per mouse overlaid with mean ± SD, n = 5–7 mice/group, ns – not significant, *** p < 0.01; **** p < 0.001. (E) Sum histopathology scores for proximal, middle, and distal colon sections from control and DSS treated WT and *Tnc^−^*^/^*^−^* mice. Data points represent scores for individual mice with bars marking the median ± interquartile range, n = 5–7 per group, *p < 0.05; **p < 0.01, (F) Scores for individual histopathology scoring parameters in distal colon segments from DSS treated WT and *Tnc^−^*^/^*^−^* mice. Data points represent scores for individual mice with bars marking the median ± interquartile range, n = 5–7 per group, ns – not significant; * p < 0.05. (For interpretation of the references to colour in this figure legend, the reader is referred to the web version of this article.)

To assess the impact of tenascin-C on the development of experimental colitis we next studied the colon architecture of DSS treated *Tnc^−^*^/^*^−^* mice (Fig. 1c). Using the colitis histopathology grading system (Table 1) the *Tnc^−^*^/^*^−^* mice displayed no histological abnormalities within the colon that may have confounded future analysis (data not shown). They also had no positive tenascin-C staining along the length of the colon, indicating that tenascin-C protein expression was ablated (Fig. 6a). The *Tnc^−^*^/^*^−^* and corresponding WT mice were imported from the Kennedy Institute of Rheumatology at the University of Oxford. Although they were on the same 129sv background as those maintained within the Roslin Institute colony and used in our initial study they were much heavier in bodyweight, possibly due to dietary influences and/or genetic drift. Optimisation experiments revealed that the 2% DSS dose did not robustly induce colitis in these mice and therefore a higher 3% DSS dose was used in all future experiments. At the end of the study on day 8 both WT and *Tnc^−^*^/^*^−^* DSS mice had a lower body weight than genotype matched controls but the body weight of *Tnc^−^*^/^*^−^* DSS mice was higher than that of DSS WT mice (Fig. 6b). A protective effect of the loss of tenascin-C was not evident using the DAI metric however with both DSS genotypes displaying a similar degree of scoring, which in both cases was significantly different from matched controls, by day eight (Fig. 6c). Colon length was similar in the control mice of both groups, but *Tnc^−^*^/^*^−^* mice appeared protected with DSS treatment resulting in significantly less colon shortening compared to DSS treated WT mice (Fig. 6d). Water consumption in all 4 groups of mice was similar (data not shown). Histological examination suggested that tissue damage was less in the *Tnc^−^*^/^*^−^* mice with greater preservation of normal morphology, such as areas of normal crypt architecture, that were largely absent in WT tissue. Quantification of these changes confirmed that the DSS treated distal colon of *Tnc^−^*^/^*^−^* mice exhibited a significantly lower pathological score compared to the WT mice. The DSS treated middle colon conversely showed no differences in score between the genotypes, potentially due to more variable pathological changes (Fig. 6e and f).

**Fig. 7:**
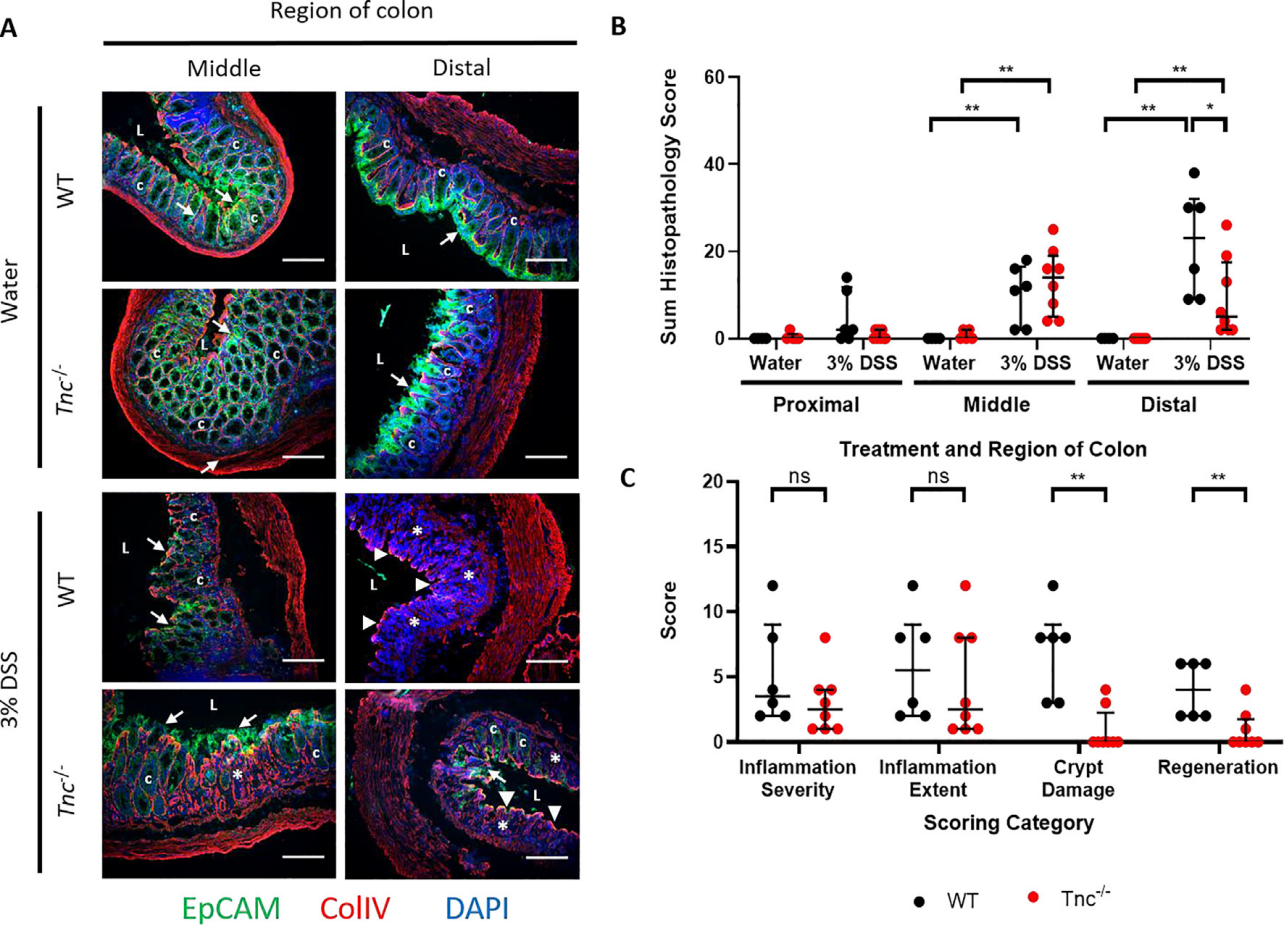
Mucosal architecture and epithelial integrity are protected in Tnc*^−^*^/^*^−^* mice treated with DSS. (A) Immunohistochemical dual staining for epithelial cells (EpCAM; green) and basement membranes (ColIV; red) of middle and distal colon sections from control and DSS treated WT and Tnc*^−^*^/^*^−^* mice. Under basal conditions epithelial cells (arrows) cover the mucosal surface with normal crypt architecture observed along the length of the colon in both WT and Tnc*^−^*^/^*^−^* mice. With DSS treatment in WT mice the mucosa is damaged with the epithelium becoming ulcerated (arrowheads) and crypt architecture abrogated (asterisks) in the middle and distal colon. In Tnc*^−^*^/^*^−^* mice with DSS treatment similar changes are observed although partial protection of the epithelium and crypt architecture is also seen. All slides were counterstained with the nuclear stain DAPI (blue). Scale bars = 250 µm. L = lumen, c = crypt. (B) Sum histopathology scores for proximal, middle, and distal colon sections from control and recovering (17 days) DSS treated WT and *Tnc^−^*^/^*^−^* mice. *Tnc^−^*^/^*^−^* mice show significantly reduced colitis scoring in the distal colon. (C) Scores for individual histopathology scoring parameters in distal colon segments from recovering DSS treated WT and *Tnc^−^*^/^*^−^* mice. *Tnc^−^*^/^*^−^* mice display less severe tissue damage compared to controls as measured by the crypt damage and regeneration categories. Data points represent scores for individual mice with bars marking the median ± interquartile range, n = 4–8 per group, ns – no significant; * p < 0.05; ** p < 0.01. (For interpretation of the references to colour in this figure legend, the reader is referred to the web version of this article.)

To identify any histological or cellular differences between the WT and *Tnc^−^*^/^*^−^* mice, which could potentially explain the differences in pathological changes observed, we first focussed on the epithelial marker, EpCAM to further assess the mucosal damage in the DSS treated WT and *Tnc^−^*^/^*^−^* mice. Colon sections from all 4 groups of mice (control, DSS treated, WT and *Tnc^−/−^)* were stained for EpCAM and type IV collagen (ColIV) to aid in delineating the structure of the mucosa. With DSS treatment, no structural changes were observed in the proximal colon of either genotype (data not shown). In contrast, in the colitic middle and distal colon of the WT mice ulceration is apparent with loss of EpCAM staining of the luminal surface of the mucosa. Furthermore, in the distal colon the damage to the mucosa resulted in the loss of its normal structure with crypts no longer obvious. In contrast, in the middle and distal colon of the DSS treated *Tnc^−^*^/^*^−^* mice while these same changes were also still present, areas of normal mucosal structure were also identified. These protected epithelial areas expressed EpCAM staining indicating that epithelial integrity was maintained. Additionally, these areas also showed preserved structural architecture with normal crypts still clearly present (Fig. 7a). We also investigated other cellular markers in WT and T*nc^−/−^* mice by immunohistochemistry, finding similar numbers and localisation of markers for macrophages (CD11c), T cells (CD3), blood vessels (CD31) and lymphatic vessels (LYVE-1) were present in the damaged mucosa of the colitic distal colon of WT and *Tnc^−^*^/^*^−^* mice (Supplementary Fig. 1, Fig. 2, Fig. 3).

To compare the ability of WT and *Tnc^−^*^/^*^−^* mice to recover from DSS induced colitis we used an adapted DSS protocol in which the mice were offered water for 17 days after the 5 days of DSS treatment and culled at day 22 (Fig. 1d). The middle and distal colon from both DSS groups still had moderate histopathological changes detectable, but similar to the acute studies the severity of the distal colon lesions were less in the *Tnc^−^*^/^*^−^* mice than in WT mice after 17 days of recovery (Fig. 7b and c).

## Discussion

In this study, the distribution of tenascin-C within the colon was altered and its expression levels were increased in DSS induced experimental colitis. The increased expression is in line with previously reported omic studies of DSS colitis, which have also reported tenascin-C upregulation in the colitic colon by immunohistochemistry, gene expression microarray and mass spectrometry [21], [36]. Additionally, tenascin-C protein has also been shown to be elevated in the colitic colons of *Il-10^−^*^/^*^−^* mice, a genetic model of IBD, suggesting this upregulation is not specific to the DSS colitis model [37]. This increase of tenascin-C in animal models is consistent with the increased tenascin-C detected in inflamed human ulcerative colitis colon tissue [20], [21]. Likewise, the altered distribution of tenascin-C noted in this study matches the disorganised tenascin-C deposition within the lamina propria of samples from patients with either ulcerative colitis or Crohn’s disease [28], [38]. Additionally, as in the DSS model, this increased tenascin-C expression has been reported to be particularly prominent within the granulation tissue stroma of ulcerations (27). The increased expression of tenascin-C in DSS treated mice occurred in parallel with the development of significant histopathological changes in the mice which, as reported by others [21], [39], developed by day six of initial DSS dosing. As such, this could suggest that tenascin-C is not an early inducer of the intestinal inflammatory response but instead only becomes increased at sites of established inflammation and tissue damage.

Besides inflammatory pathology, the IBDs are also associated with fibrosis such as thickening of muscularis mucosa which is observed in both ulcerative colitis [40] and Crohn’s disease [41]. As shown in this study, and elsewhere [42], DSS colitis replicates this muscularis mucosal thickening. With tenascin-C’s localization to the muscularis mucosa and its known pro-fibrotic properties [43] this potentially implicates tenascin-C in another aspect of pathological changes in IBD. Indeed, findings from patients with the less common microscopic collagenous colitis may provide additional evidence for this pro-fibrotic hypothesis. Collagenous colitis is characterized by an absence of gross colonic pathology, such as ulceration, with only the presence of histological inflammation alongside the deposition of a thick subepithelial collagen band [44]. This subepithelial collagen band coincides with the localization of the basal apical mucosal tenascin-C staining which is shown to be significantly increased in collagenous colitis patients [45]. As such, this again places tenascin-C at a site of colonic inflammatory associated fibrosis suggesting further its involvement. Together these observations imply that tenascin-C’s upregulation in colitis is conserved across disease models and types as well as species, implicating it in a common colitis promoting role.

The mechanisms by which tenascin-C expression is induced may involve the microbiome, which has been shown to be essential for the establishment of DSS colitis [46]. Early in DSS dosing, colonic permeability increases significantly, resulting in luminal bacterial content leaking into the underlying lamina propria [47]. A variety of bacterial components acting via different PRR, including TLR 1, 2, 4, and 5, have previously been shown to drive tenascin-C production in human dendritic cells (24). An endogenous mechanism may also potentially be responsible with the observation that the earliest observed pathological sign was limited to infiltration of inflammatory cells seen on day four of DSS treatment. These cells were mainly mononuclear in the early stages but predominantly neutrophils once the epithelial barrier is breached. This differs from reports that neutrophils are the earliest immune cell type recruited to the colon in DSS colitis [48] but the difference could be due to the lack of epithelial damage present in the early stages in the present study. Neutrophils display limited ability to produce tenascin-C and thus are unlikely the source of the tenascin-C subsequently found at day six [49]. However, neutrophils can produce significant amounts of TNF [50] which in turn has been shown to induce the expression of tenascin-C in a number of cells types [51], [52].

Observations of the *Tnc^−^*^/^*^−^* mouse have established that tenascin-C protein is not required for normal development [35], [53] but does have a role in the inflammatory response [54]. From the study of various animal models it has been shown that while an acute response can still be initiated the loss of tenascin-C proves protective of progression to more serious chronic disease (18). In these studies the protection in the *Tnc^−^*^/^*^−^* mouse was attributed to tenascin-Cs ability to drive the production of pro-inflammatory cytokines as well as the induction of pathological Th1 and Th17 T cell responses resulting in more severe disease in its presence [55], [56], [57]. In contrast, there are other reports showing that the lack of tenascin-C perturbed tissue repair in response to the initial insult, resulting in a more severe inflammatory response [58], [59], [60], [61], [62]. Despite tenascin-Cs known association with IBD [20], [28], [63], the gastrointestinal system is one of the few remaining systems in which tenascin-C’s role in inflammatory pathology is unclear.

**Fig. 8:**
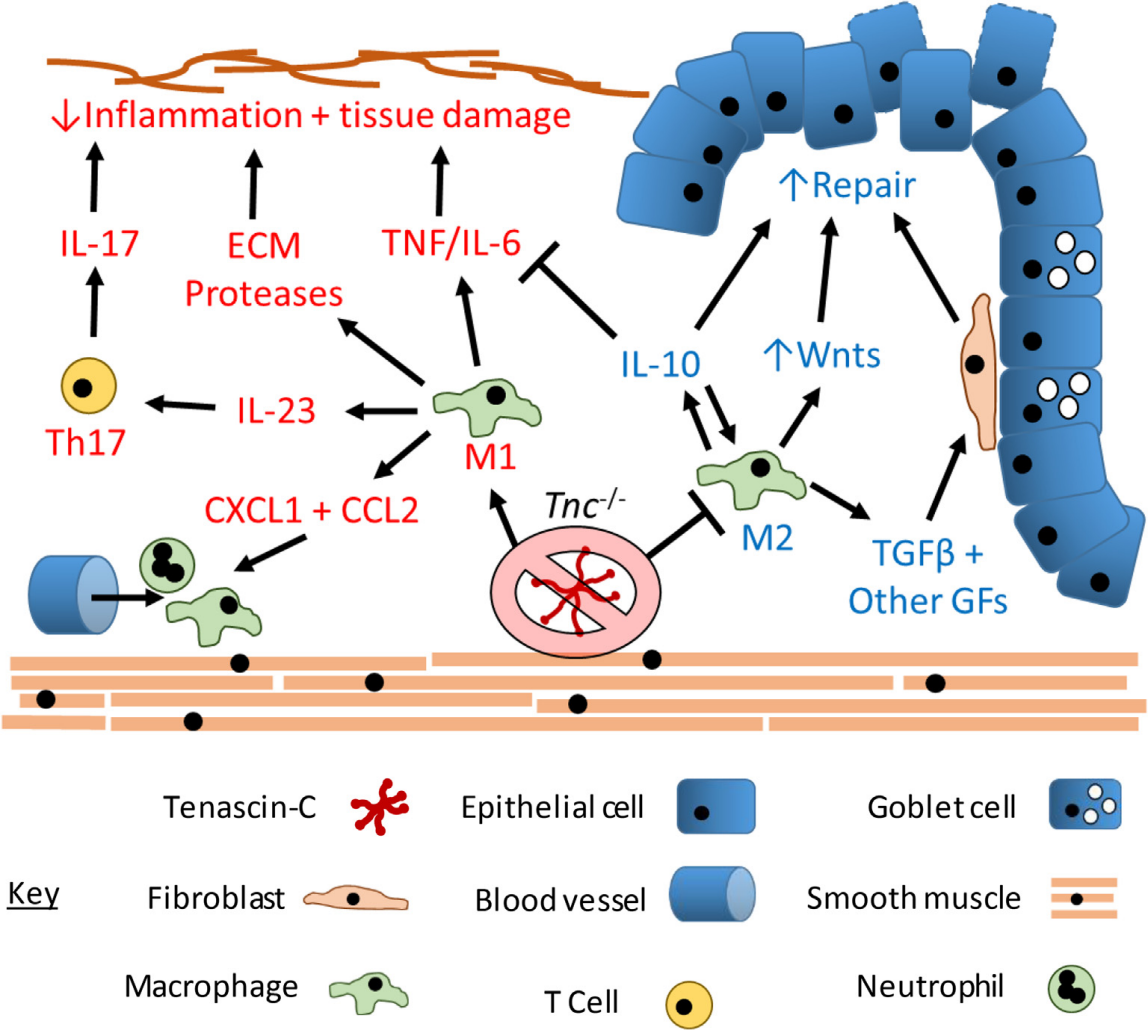
Summary model of potential protective mechanisms of genetic ablation of tenascin-C in DSS colitis. Genetic ablation of tenascin-C results in downregulation of pro-inflammatory processes (red) and promotion of anti-inflammatory and pro-repair processes (blue). Tenascin-C engages pro-inflammatory receptors on macrophages and stromal cells to drive pro-inflammatory cytokine and ECM protease production. These cytokines drive the pathological inflammatory process including promoting pathological adaptative immune processes such as the differentiation of IL-17 producing Th17 T cells. Tenascin-C also drives further immune cell recruitment to the colon via promoting the production of chemokines such as CXCL1 and CCL2. These processes all contribute to the excessive tissue damaging inflammatory response and are mitigated by tenascin-Cs loss in *Tnc^−^*^/^*^−^* mice. Furthermore, loss of tenascin-C results in the promotion of anti-inflammatory immune responses such as the polarisation of macrophages to an M2 phenotype. These cells produce the potent anti-inflammatory cytokine IL-10 which inhibits inflammatory processes such as pro-inflammatory cytokine production and promotes epithelial repair. M2 macrophages additionally promote tissue repair by secretion of a variety of other growth factors such as TGFβ and Wnts which act of stromal and epithelial cells to promote tissue regeneration. (For interpretation of the references to colour in this figure legend, the reader is referred to the web version of this article.)

The data from this present study revealed that *Tnc^−^*^/^*^−^* mice exhibited a partial protection against IBD with a lower pathological score. These observations are consistent with other data where neutralization of TNF activity or ablation of IL-6 expression in DSS treated mice attenuated colonic injury and inflammatory cell infiltration [64], [65]. Furthermore, therapies targeting TNF and IL-6 have proven clinical efficacy in the treatment of IBD [66], [67]. The reduced levels of inflammatory cell infiltration may be a result of altered CXCL1, CXCL2, and CCL2 chemokine levels which are reduced in *Tnc^−^*^/^*^−^* mice [68], [69]. All of these chemokines have previously been shown to be increased in DSS colitis [70] and are also increased in IBD patients [71], [72] suggesting they are relevant to IBD pathogenesis. Furthermore, the membrane bound extracellular protease MMP14 has been implicated in the pathogenesis of IBD and thus with tenascin-C’s known link to its expression shows another potential mechanism by which tenascin-C deficiency may be protective in colitis [73]. A summary of the proposed protective mechanisms in DSS colitis is presented in Fig. 8 highlighting the possible anti-inflammatory and pro-repair affects that are mediated in the absence of tenascin-C.

Having shown a protective effect of loss of tenascin-C during the acute phase of the DSS model the observation period was extended to study the impact of tenascin-C on colitis resolution. This was undertaken as tenascin-C, as well as having known roles in supporting the initial inflammatory responses, has also been shown to have roles in the later repair phases (12). The reduced level of histopathological changes noted in the *Tnc^−^*^/^*^−^* mice may simply be a result of a reduction in initial inflammation and thus damage in the knockouts however it cannot be ruled out that the loss of tenascin-C is not positively impacting the wound healing response as well. In contrast, a previous report proposed tenascin-C as a protective factor in DSS colitis which mediated epithelial healing [74]. The reason(s) for the different DSS response between the two studies is unclear but may be related to the sex and genetic background of the mice and also to the presence of a recovery period after the withdrawal of the DSS. Similarly, data on dermal wound healing models have implicated tenascin-C in promoting keratinocyte proliferation and migration [75]. The findings in the current study of reduced tissue damage, including reduced ulceration, in *Tnc^−^*^/^*^−^* mice however would suggest that this is not the case in the gut.

The results from this study show that tenascin-C has a central role in the etiology of IBD and, whilst further work is required, they provide a platform to investigate the possibility of using tenascin-C neutralizing antibodies to reduce the inflammatory pathology and tissue damage associated with IBD and other inflammatory disorders.

## Funding

JO was funded by a scholarship from Medical Research Scotland (PhD-771-2014). CF, GM and LAS were supported by the Biotechnology and Biological Sciences Research Council (BBSRC) via an Institute Strategic Programme Grant (BB/J004316/1). KSM was funded by Versus Arthritis Senior Research Fellowship (20003). For the purpose of open access, the author has applied a CC BY public copyright licence to any Author Accepted Manuscript version arising from this submission.

## Declaration of Competing Interest

The authors declare that they have no known competing financial interests or personal relationships that could have appeared to influence the work reported in this paper.
