# Supplementary material for: Tenascin-C is a driver of inflammation in the DSS model of colitis
Source: Matrix Biol Plus. 2022 May 23;14:100112. doi: 10.1016/j.mbplus.2022.100112 (PMC9166467; doi:10.1016/j.mbplus.2022.100112)
Supplement: Supplementary data 1 [file mmc1.docx]

| Mouse ID: |  |  | | | | | | PPL # | | | |
| --- | --- | --- | --- | --- | --- | --- | --- | --- | --- | --- | --- |
| 1. ***Date:*** |  |  |  |  |  |  |  |  | | | |
| 1. *APPEARANCE* |  |  |  |  |  |  |  |  | | | |
| Coat lost sheen |  |  |  |  |  |  |  |  | | | |
| Coat staring |  |  |  |  |  |  |  |  | | | |
| Hair loss |  |  |  |  |  |  |  |  | | | |
| Failure to groom |  |  |  |  |  |  |  |  | | | |
| Discharge from eyes or nose |  |  |  |  |  |  |  |  | | | |
| Hunched body |  |  |  |  |  |  |  |  | | | |
| Change in gait |  |  |  |  |  |  |  |  | | | |
| 1. *BEHAVIOR* |  |  |  |  |  |  |  |  | | | |
| Change in temperament |  |  |  |  |  |  |  |  | | | |
| Isolated, away from others |  |  |  |  |  |  |  |  | | | |
| Restless |  |  |  |  |  |  |  |  | | | |
| Reluctant to move |  |  |  |  |  |  |  |  | | | |
| Recumbent |  |  |  |  |  |  |  |  | | | |
| *DSS SYMPTOMS* |  |  |  |  |  |  |  |  | | | |
| Stool consistency |  |  |  |  |  |  |  |  | | | |
| Fecal blood |  |  |  |  |  |  |  |  | | | |
| 1. *CLINICAL SIGNS* |  |  |  |  |  |  |  |  | | | |
| Weight (g) |  |  |  |  |  |  |  |  | | | |
| Cold to touch |  |  |  |  |  |  |  |  | | | |
| Breathing abnormal |  |  |  |  |  |  |  |  | | | |
|  |  |  |  |  |  |  |  |  | | | |
| Initials |  |  |  |  |  |  |  |  | | | |
| Date |  |  |  |  |  |  |  |  | | | |
| Place numerical score in appropriate box (see below)  If there is cause for concern, inform the NACWO, NVS and/or the PPL holder and record details in “Animal Room Log Book”. | | | | | | | | |  |  |  |

**Score System**

Normal (no fecal blood/normal well-formed stools) = 0

Slightly abnormal (blood visible in stool/soft pasty stool) = 1

Significant change (gross rectal bleeding/diarrhoea liquid stool) = 2

Euthanasia will be performed when any of the following criteria are met:

1. A score of 2 is given to 3 or more parameters (except for “clinical signs”)
2. A total score of 5 is reached from any parameter
3. Either clinical sign – cold to touch or abnormal breathing are observed.
4. An overall weight loss of 20% is observed

Supplementary Table 1:

| **Target** | **Antibody Name** | **Supplier** | **Species and isotype** | **Working concentration** |
| --- | --- | --- | --- | --- |
| Tenascin-C | MTn-12 | Sigma-Aldrich | Rat monoclonal IgG1 | 1µg/ml |
| CD3 | 17A2 | Biolegend | Rat monoclonal IgG2b | 10µg/ml |
| CD11c | HL3 | Biolegend | Armenian Hamster monoclonal IgG | 10µg/ml |
| CD31 | MEC13.3 | Biolegend | Rat monoclonal IgG2a | 1µg/ml |
| CD326 | G8.8 | Biolegend | Rat monoclonal IgG2a | 1µg/ml |
| LYVE-1 | ab14917 | Abcam | Rabbit polyclonal | 10µg/ml |
| Collagen IV | ab6586 | Abcam | Rabbit polyclonal | 1µg/ml |

Supplementary Table 2: List of primary antibodies used.

| **Target** | **Antibody Name** | **Supplier** | **Conjugate** | **Working concentration** |
| --- | --- | --- | --- | --- |
| Anti-rat IgG | A-11006 | Sigma-Aldrich | Alexa Fluor 488 | 2µg/ml |
| Anti-rat IgG | 112-065-167 | Jackson Immunoresearch | Biotin | 2µg/ml |
| Anti-rat IgG1 | MRG1-58 | Biolegend | FITC | 2µg/ml |
| Anti-rat IgG2a | MRG2a-83 | Biolegend | Alexa Fluor 594 | 2µg/ml |
| Anti-rat IgG2b | MRG2b-85 | Biolegend | Alexa Fluor 594 | 2µg/ml |
| Anti-rabbit IgG | A-11012 | Biolegend | Alexa Fluor 594 | 2µg/ml |
| Anti-Armenian hamster IgG | Poly4055 | Biolegend | Alexa Fluor 594 | 2µg/ml |
| Streptavidin | 405240 | Biolegend | Alexa Fluor 594 | 2µg/ml |

Supplementary Table 3: List of secondary antibodies used.


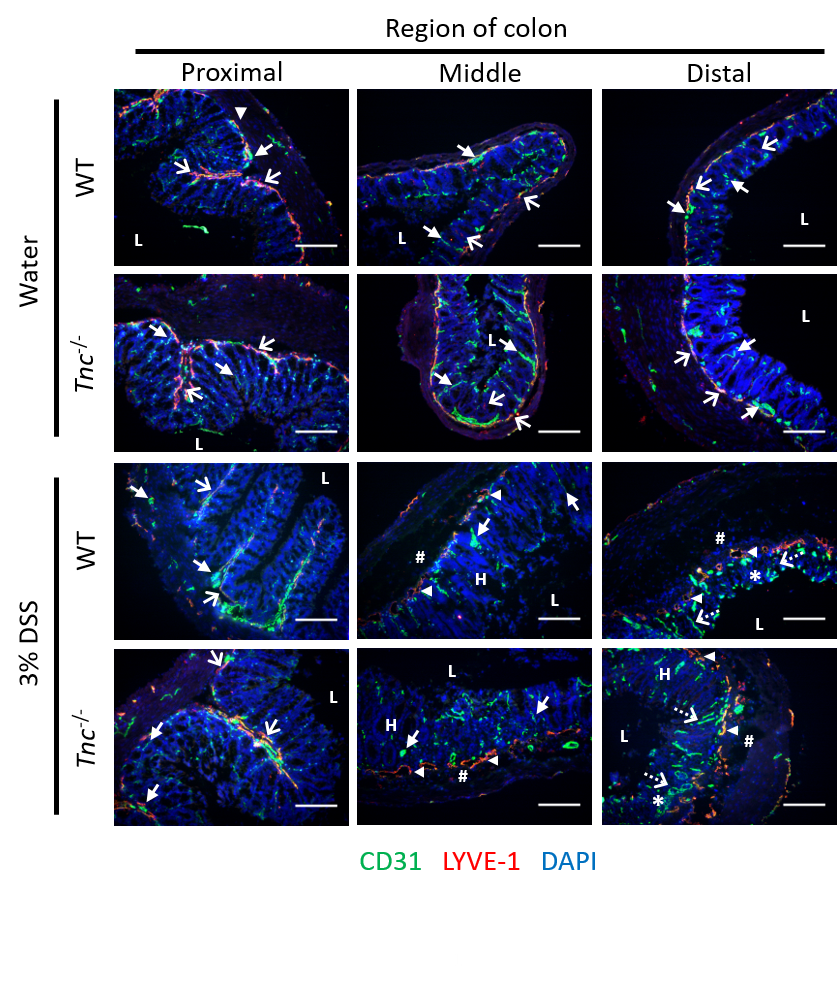


**Supplemental Figure 1.** Number and distribution of blood and lymphatic vessels are similar between WT and *Tnc*^-/-^ mouse colons under resting or colitic conditions. Dual staining for blood (CD31; green) and lymphatic (LYVE-1; red) vessels of proximal, middle, and distal colon sections from control and 3% DSS dosed WT and *Tnc*^-/-^ mice. Blood (closed arrows) and lymphatics (open arrows) in the mucosa and submucosa appear normal in both genotypes under basal conditions. Colitis results in an upregulation of blood vessels in the damaged mucosa (dashed arrows) and dilated lymphatics (arrowheads) alongside submucosal oedema (hash) in the middle and distal colon of both genotypes. All slides were counterstained with the nuclear stain DAPI (blue) and imaged using a Leica DMLB microscope with a 10x objective Scale bars = 250µm. L = lumen, * = mucosal granulation tissue, H = hyperplastic epithelium.


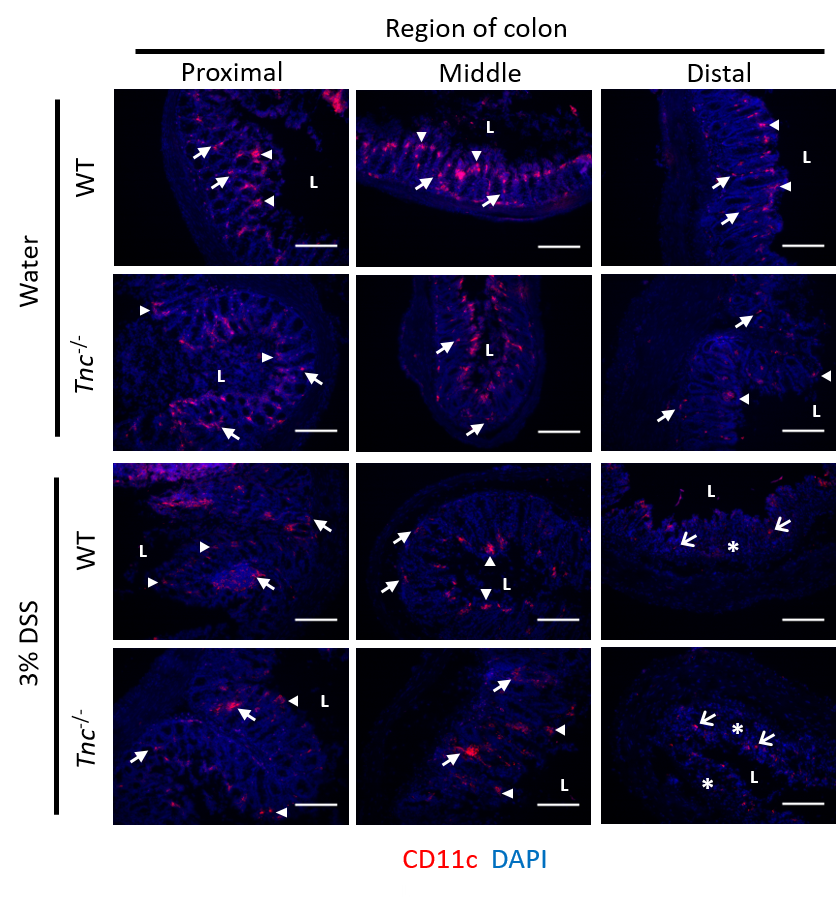


**Supplemental Figure 2.** Colonic tissue resident macrophage localisation is unaffected by loss of tenascin-C. Staining for colonic tissue resident macrophages (CD11c; red) of proximal, middle, and distal colon sections from control and 3% DSS dosed WT and *Tnc*^-/-^ mice. Macrophages (closed arrows) are found throughout the lamina propria, including at apical subepithelial sites (arrowheads), along the length of the colon in both genotypes. Under colitic conditions similar numbers of resident macrophages are found within the damaged mucosa (open arrows). All slides were counterstained with the nuclear stain DAPI (blue) and imaged using a Leica DMLB microscope with a 10x objective Scale bars = 250µm. L = lumen, * = damaged mucosa


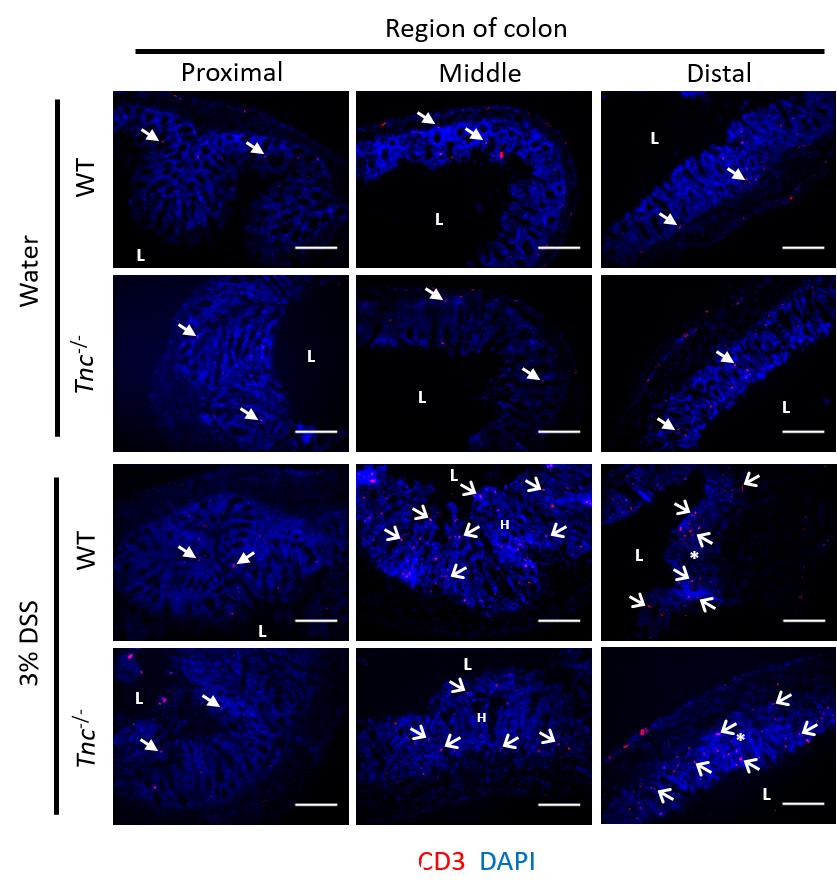


**Supplemental Figure 3** Cell infiltration of the colitic colon is observed in both WT and *Tnc*^-/-^ mice. Staining for T cells (CD3; red) of proximal, middle, and distal colon sections from control and 3% DSS dosed WT and *Tnc*^-/-^ mice. Under basal conditions low numbers of T cells (closed arrows) are found within the lamina propria and muscularis externa along the length of the colon in both genotypes. Likewise, in both genotypes with DSS dosing elevated numbers of T cells are observed infiltrating the damaged mucosa of the middle and distal colon (open arrows). All slides were counterstained with the nuclear stain DAPI (blue) and imaged using a Leica DMLB microscope with a 10x objective Scale bars = 250µm. L = lumen, * = damaged mucosa, H = hyperplastic epithelium.
